# Single-Cell DNA Barcoding Using Sequences from the Small Subunit rRNA and Internal Transcribed Spacer Region Identifies New Species of *Trichonympha* and *Trichomitopsis* from the Hindgut of the Termite *Zootermopsis angusticollis*

**DOI:** 10.1371/journal.pone.0058728

**Published:** 2013-03-11

**Authors:** Vera Tai, Erick R. James, Steve J. Perlman, Patrick J. Keeling

**Affiliations:** 1 Department of Botany, University of British Columbia, Vancouver, British Columbia, Canada; 2 Department of Biology, University of Victoria, Victoria, British Columbia, Canada; Université Paris Sud, France

## Abstract

To aid in their digestion of wood, lower termites are known to harbour a diverse community of prokaryotes as well as parabasalid and oxymonad protist symbionts. One of the best-studied lower termite gut communities is that of *Zootermopsis angusticollis* which has been known for almost 100 years to possess 3 species of *Trichonympha* (*T. campanula*, *T. collaris*, and *T. sphaerica*), 1 species of *Trichomitopsis* (*T. termopsidis),* as well as smaller flagellates. We have re-assessed this community by sequencing the small subunit (SSU) rRNA gene and the internal transcribed spacer (ITS) region from a large number of single *Trichonympha* and *Trichomitopsis* cells for which morphology was also documented. Based on phylogenetic clustering and sequence divergence, we identify 3 new species: *Trichonympha postcylindrica*, *Trichomitopsis minor*, and *Trichomitopsis parvus* spp. nov. Once identified by sequencing, the morphology of the isolated cells for all 3 new species was re-examined and found to be distinct from the previously described species: *Trichonympha postcylindrica* can be morphologically distinguished from the other *Trichonympha* species by an extension on its posterior end, whereas *Trichomitopsis minor* and *T. parvus* are smaller than *T. termopsidis* but similar in size to each other and cannot be distinguished based on morphology using light microscopy. Given that *Z. angusticollis* has one of the best characterized hindgut communities, the near doubling of the number of the largest and most easily identifiable symbiont species suggests that the diversity of hindgut symbionts is substantially underestimated in other termites as well. Accurate descriptions of the diversity of these microbial communities are essential for understanding hindgut ecology and disentangling the interactions among the symbionts, and molecular barcoding should be a priority for these systems.

## Introduction

Termites harbour a diverse community of microbial symbionts in their hindguts, many of which aid the digestion of lignocellulose. The “lower” termites (Mastotermitidae, Kalotermitidae, Termopsidae, Hodotermitidae, Serritermitidae, and Rhinotermitidae) host both protist and prokaryotic symbionts, while the “higher” termites (Termitidae) host only prokaryotes [1]. The protists found in lower termites are primarily Parabasalia and Oxymonadida [2] and most of the known diversity of both lineages resides in the hindguts of termites. The composition of the hindgut community is generally species-specific: each termite species has its own set of symbionts, but related termites share a similar community of related symbionts, altogether indicating some degree of co-evolution between symbionts and termite hosts [3].

The identification and classification of termite hindgut symbionts has a history extending over more than 100 years and has largely been based on morphological criteria. Molecular characterization has been applied slowly, in part because almost none of the symbionts have been brought into culture. Molecular sequencing of the small subunit (SSU) rRNA gene from manually isolated cells has only recently begun to substantially supplement the morphological characterization, but the resulting molecular phylogenetic analyses have already contributed a great deal to clarifying taxonomy, in particular with respect to parabasalians [4]–[11]. Sequencing of SSU rRNA genes from the hindgut community followed by fluorescent in situ hybridization has also linked molecular data to the morphologically described species [12]–[14].

In general, molecular data have been used to test hypotheses concerning the evolution of established species [15]–[19] or to formally describe new symbiont species from termites that have not been investigated previously using classical criteria [20], [21]. Another question of equal importance, however, is whether the classical morphology-based descriptions of termite hindgut communities can be validated using molecular markers. Despite the obvious utility of molecular data to test the defined compositions of these communities, they have seldom been tested specifically (but see Strassert et al. 2009). This is unfortunate, because an accurate description of a hindgut community is a basic first step to understanding its ecology, the interactions between the biota, the evolution of hindgut symbionts, and the factors influencing community composition.

Here we specifically test the seemingly well-known composition of the protist community in the hindgut of the Pacific Dampwood termite, *Zootermopsis angusticollis.* This community has been studied for nearly 100 years, and since the early 1900 s it has been documented to contain seven species of protist: the parabasalians *Hexamastix termopsidis*, *Tricercomitus termopsidis*, *Trichomitopsis termopsidis*, *Trichonympha campanula*, *Trichonympha collaris*, and *Trichonympha sphaerica*, and the oxymonad *Streblomastix strix* [22]–[29]. These same protist species are also found in the hindgut of *Z. nevadensis,* the closest relative of *Z. angusticollis*. This community has been re-visited in a variety of studies, including some of the earliest molecular characterization studies [4], [6], [30] and metatranscriptomic analyses of protist hindgut symbionts [31], [32], making it arguably the best-studied community of any lower termite.

By characterizing sequences from the SSU rRNA gene and internal transcribed spacer (ITS) region from over 50 manually isolated cells of the largest symbionts from the *Z. angusticollis* hindgut, we find that even the species-level diversity of this well-studied community has been substantially underestimated. Rather than three species of *Trichonympha* we find there are four, and rather than one species of *Trichomitopsis* we find there are three, almost doubling the number of large and most easily identifiable species. Interestingly, all three new species found by molecular characterization also correlate with morphological variation. This expansion of characterized diversity of the largest symbionts within such a well-studied host termite suggests that parabasalian symbiont diversity as a whole may be even more significantly underestimated and a detailed characterization of symbiont diversity at the molecular level should be a first step in tackling hindgut ecology and symbiont interactions in any other model termite.

## Results and Discussion

To test whether our long-established understanding of the hindgut community composition of *Z. angusticollis* is correct, 77 single cells representing distinct morphotypes of *Trichonympha* and *Trichomitopsis* were manually isolated, photographed, and characterized by DNA sequencing. The termites from which these hindgut symbionts were isolated were confirmed to be *Z. angusticollis* as their mitochondrial cytochrome oxidase I (COI) gene sequences (GenBank accession KC136610 and KC136611) were identical to those previously sequenced from *Z. angusticollis* [33].

### Diversity of *Trichonympha* in *Z. angusticollis*

**Figure 1:**
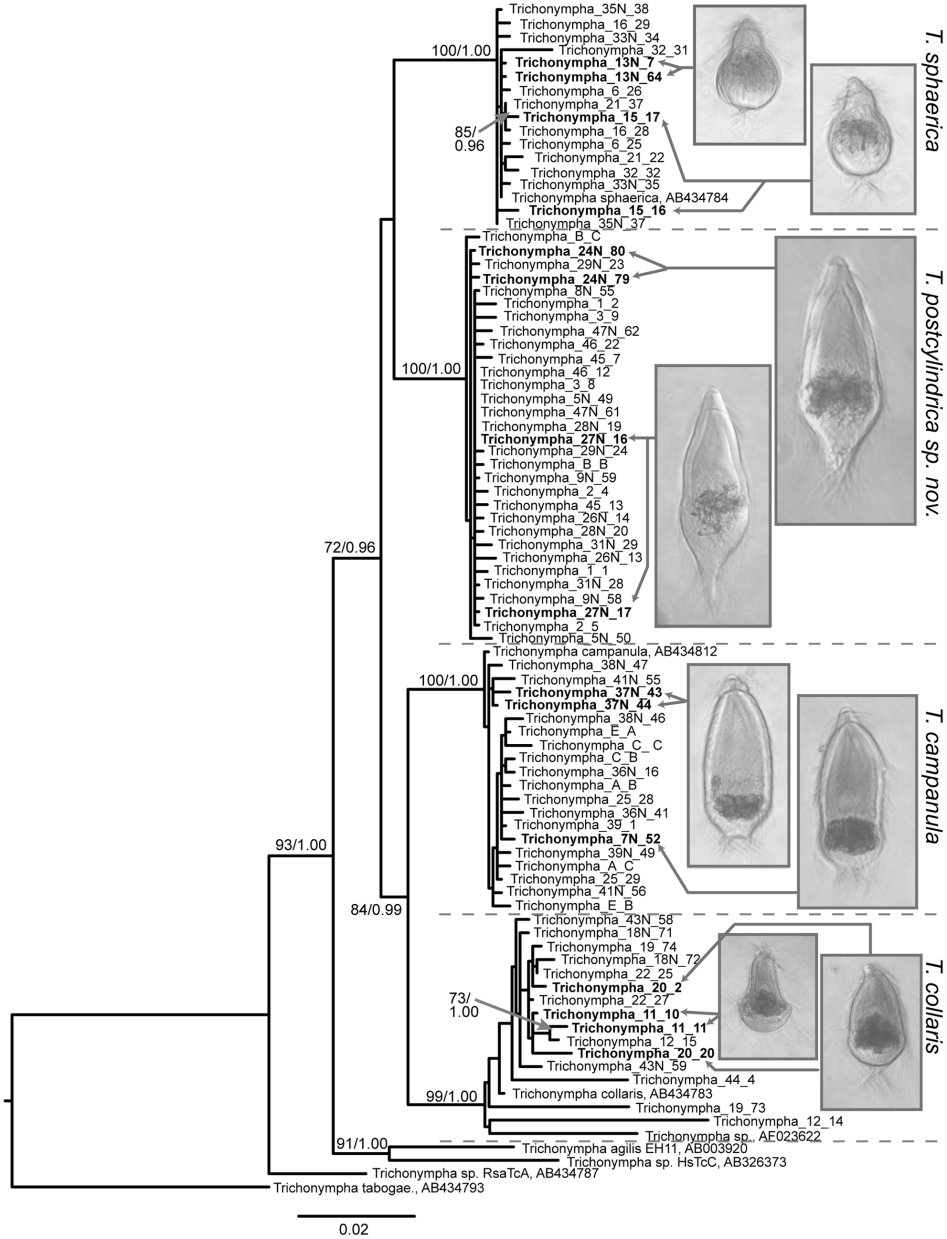
Phylogenetic tree of single-cell SSU rRNA barcodes from *Trichonympha* isolated from the hindgut of *Zootermopsis angusticollis*. Images are examples of manually isolated cells with arrows pointing to the SSU sequences obtained from these single cells. SSU rRNA sequences from *Trichonympha* species from *Zootermopsis nevadensis* were included for reference (*T. sphaerica*  =  AB434784, *T. campanula*  =  AB434812, and *T. collaris*  =  AB434783). AF023622 is from *T. collaris* isolated from the hindgut of *Z. angusticollis*. Also included are representatives of the next most closely related SSU rRNA sequences available: *T. agilis* from *Reticulitermes speratus*  =  AB003920, *T. sp.* from *Hodotermopsis sjoestedti*  =  AB326373, *T. sp.* from *Reticulitermes santonensis*  =  AB434787, *T. tabogae* from *Incisitermes tabogae*  =  AB434793. The best ML tree is shown. Numbers at nodes indicate ML bootstrap support and Bayesian posterior probability values. Statistical support is shown only for nodes with >70% bootstrap support and >0.90 posterior probability.

**Figure 2:**
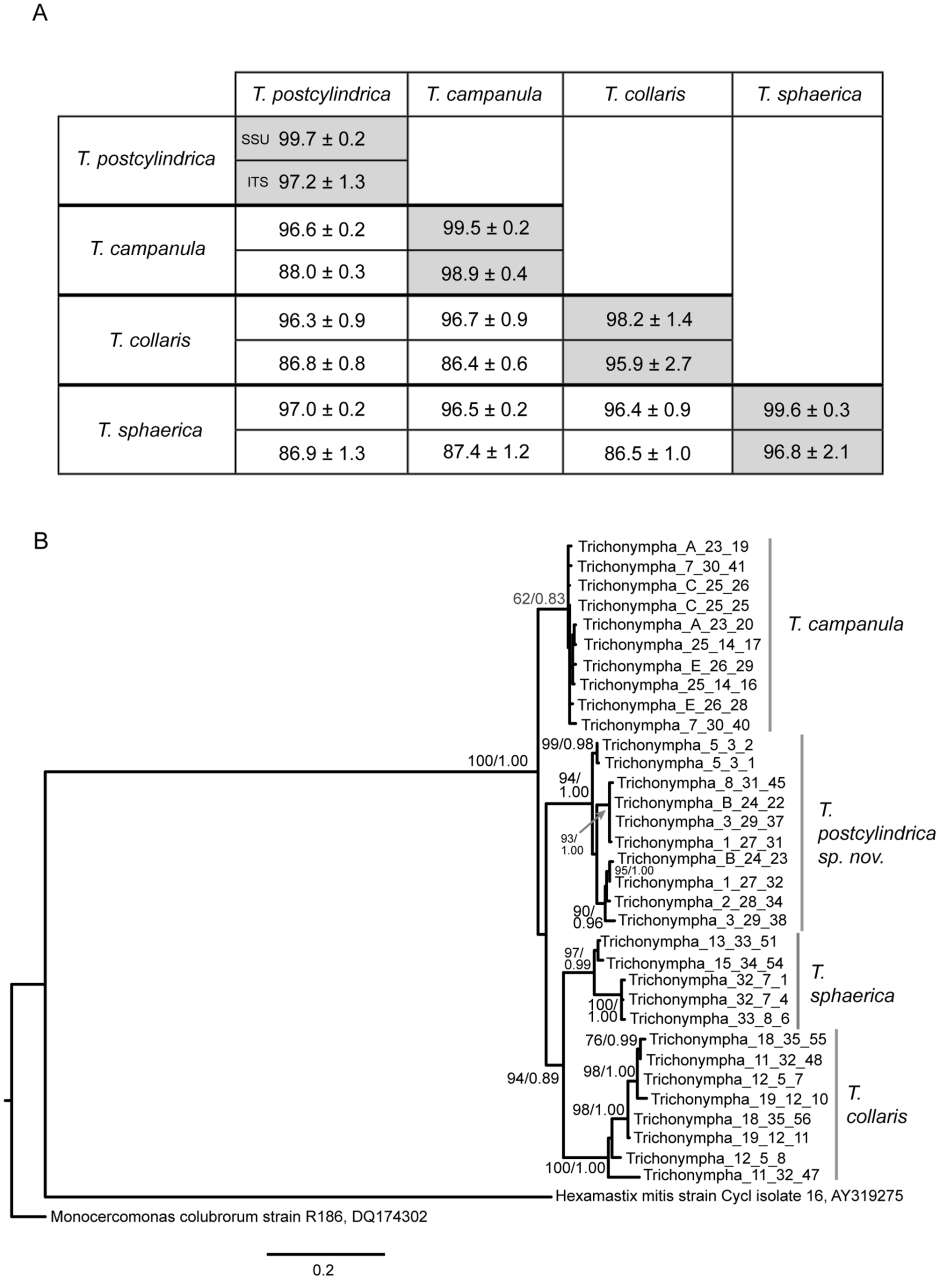
Comparing SSU and ITS barcodes from manually isolated *Trichonympha* cells from *Zootermopsis angusticollis.* A) Pairwise similarity matrix for DNA sequences from *Trichonympha* species. The upper and lower values are for SSU and ITS sequences, respectively. The mean pairwise similarities (± standard deviation) for comparisons of sequences within and between species are reported. B) Phylogenetic tree of single-cell ITS barcodes from *Trichonympha* isolated from the hindgut of *Zootermopsis angusticollis*. Sequences from *Hexamastix mitis* and *Monocercomonas colubrorum* were included to root the tree. The best ML tree is shown. Numbers at nodes indicate ML bootstrap support and Bayesian posterior probability values. Statistical support is shown only for nodes with >70% bootstrap support and >0.90 posterior probability. Statistical support for the *T. campanula* node (in italics) is also shown which does not satisfy the above criteria.

52 single cells matching the overall description of *Trichonympha* were manually isolated from *Z. angusticollis*. After purification of their DNA and PCR amplification, nearly 1500 bp of the SSU rRNA gene from 42 single cells were successfully sequenced. In phylogenetic analyses including these new sequences together with existing *Trichonympha* homologues, the majority of the new sequences clustered with *T. campanula* (clones from ten cells), *T. collaris* (clones from eight cells), and *T. sphaerica* (clones from eight cells) sequences previously characterized from the closely related host, *Z. nevadensis* (Figure 1). The morphology of the cells from which these clones were derived also matched the expected morphology of these species (Figure 1), and no cell yielded clones that fell into different clusters, as expected. However, sequences from 16 isolated cells formed a fourth lineage as distinct from other *Zootermopsis Trichonympha* species as they are from one another (Figure 1). Within this cluster, the mean pairwise identity (± standard deviation) was 99.7±0.2%, whereas the level of similarity between sequences from the new cluster and those of its nearest neighbor (*T. sphaerica*) fell to only 97.0±0.2% identity (Figure 2A). These values are similar to within- and between-species pairwise comparisons for the established species *T. campanula and T. sphaerica*. SSU sequences from *T. collaris* exhibited the greatest within-species diversity. Most of the sequences in the *T. collaris* cluster were nearly identical to the *Z. nevadensis T. collaris*, but four sequences formed long-branches that lowered the average within-species similarity to 98.2±1.4% (Figure 2A). Interestingly, one of these was a *T. collaris* sequence previously characterized from *Z. angusticollis* (AF023622) [4] which was not highly similar to any characterized here.

The coherence of this new cluster was also tested by analyzing the ITS region, which is typically a much more divergent marker. There is little ITS data available for parabasalians and these data are the first for the Trichonymphea. The ITS region was successfully amplified from 19 of the *Z. angusticollis Trichonympha* cells from which SSU rRNA was sequenced. Once again, the phylogenetic analyses of the ITS sequences showed four distinct clusters with the individual cells corresponding exactly to the SSU rRNA clusters (Figure 2B). The mean within-cluster similarity was over 95.9% for all four clusters, whereas the mean between-cluster similarity was 88.0% or less (Figure 2A). *T. collaris* again shows a higher within-species diversity than the others due to a couple of divergent sequences (one from the same cell as a less divergent copy). It is common for protist cells to have divergent rRNA gene sequences as their genomes contain multiple copies of the rRNA operon, but the range of diversity within a species or in this case within a cell is an important consideration as molecular barcodes to identify protist species and other sequence-based diversity estimates are more widely applied [34], [35].

The branching order of the four *Trichonympha* clusters was different in the SSU and ITS trees, but neither topology was rejected based on an approximately unbiased (AU) test of all possible topologies (Table S1). The order in which these species originated may be difficult to determine if the multiple species of *Trichonympha* radiated in a relatively short period of time.

**Figure 3:**
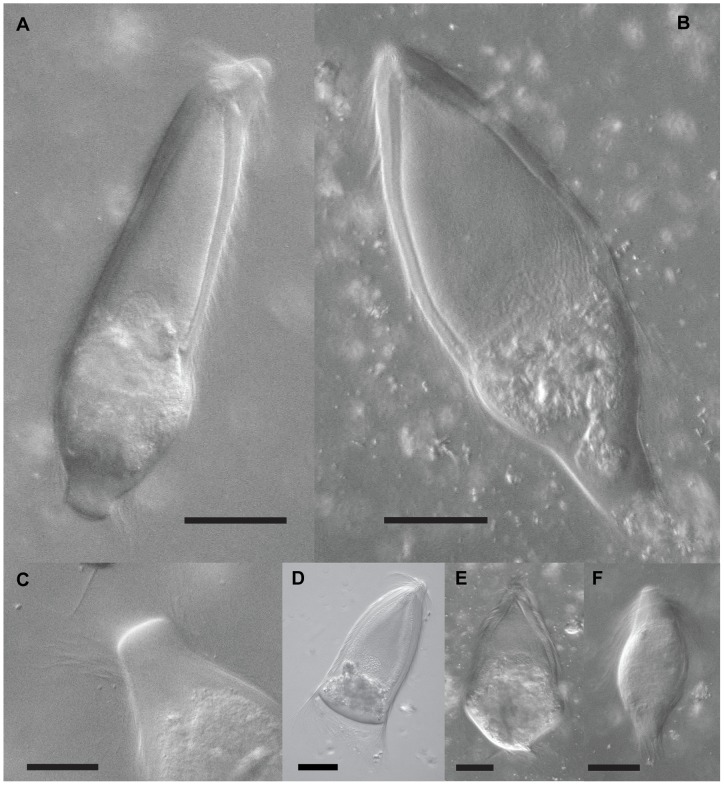
Differential interference contrast (DIC) light micrographs of *Trichonympha* species in the hindgut of *Zootermopsis angusticollis*. (A & B) Two cells matching the morphological description of *T. postcylindrica sp. nov.* These very large *Trichonympha* cells average 180 µm in length and are distinguished from other *Trichonympha* species in *Z. angusticollis* primarily by a posterior cylindrical projection (see detail in C). Also visible is a single posterior nucleus and a distinctly non-granular ectoplasmic region at the periphery of the anterior flagellated zone. (D–F) DIC micrographs for comparison of the three previously recognized species of *Trichonympha* in *Z. angusticollis*, (D) *T. campanula* (note the larger length:width ratio compared to *T. collaris*, posterior nucleus, and nondescript posterior end, (E) *T. collaris* (note the smaller length:width ratio and less posterior nucleus compared to *T. campanula*), and (F) *T. sphaerica* (note the smaller size, spherical cell shape, and anterior nucleus). All scale bars are 50 µm.

As stated above, the morphology of the cells from which *T. campanula, T. collaris, and T. sphaerica* sequences were characterized corresponded to the known morphology of these symbiont species (Figure 1, Figure 3D–F). We therefore examined the morphology of the 16 isolated cells from which all sequences falling into the fourth cluster were derived. These were similar in size and morphology to *T. campanula* averaging 180 µm×67 µm with an average length to width ratio of 2.7. These cells also consistently presented an extension at the posterior end (Figure 1, Figure 3A–C) that is not formally a defining character of *T. campanula* or any other *Trichonympha* species in *Zootermopsis*. Interestingly, in the formal description of *T. campanula*, Kofoid and Swezy remarked on the variation in morphology of the posterior end of *Trichonympha* cells [24], even describing cylindrical extensions on the posterior end that are identical to the ones we observed, but they did not consider this morphotype to be a distinct species. Based on this morphological characteristic and on the distinct clusters formed by these cells in analyses of molecular diversity from single cells, we have named this species *Trichonympha postcylindrica sp. nov.* (see Taxonomic Synopsis below).

### Diversity of *Trichomitopsis* in *Z. angusticollis*

**Figure 4:**
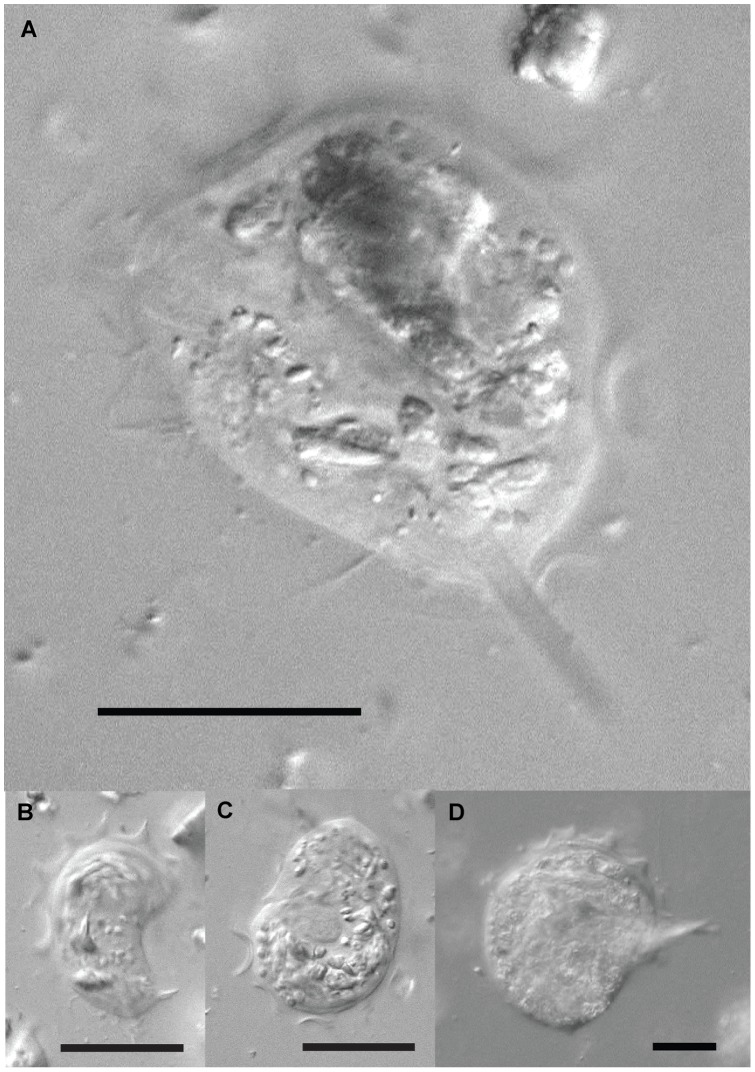
Differential interference contrast (DIC) light micrographs of *Trichomitopsis* morphotypes in the hindgut of *Zootermopsis angusticollis*. (A–C) Smaller morphotypes of *Trichomitopsis* (approximately 25 µm in diameter) corresponding to either *T. parva* or *T. minor*, which at present can only be distinguished from one another using molecular data. (D) *Trichomitopsis termopsidis* which is distinguishable by its much larger size. All scale bars are 20 µm.

The second large and distinctive genus in *Zootermopsis* is the trichomonad *Trichomitopsis*. Kofoid and Swezy [23] noted size variation in *Trichomitopsis termopsidis* ranging from 16 to over 200 µm in length, but did not consider that multiple species comprise this morphotype. It is thought that the very large cells were about to go through multiple fission events resulting in many smaller cells [23]. We did not observe the extremes of this size range, but did observe two distinct sizes of *Trichomitopsis* in the hindguts of *Z. angusticollis* (Figure 4). The larger cells were approximately 50 µm in diameter, whereas the smaller cells were approximately 25 µm in diameter. Five larger and 20 smaller cells were collected individually and the SSU rRNA was sequenced successfully from 3 larger and 10 smaller single cells. Of these, ITS sequence data was also obtained from 3 and 7 cells, respectively.

**Figure 5:**
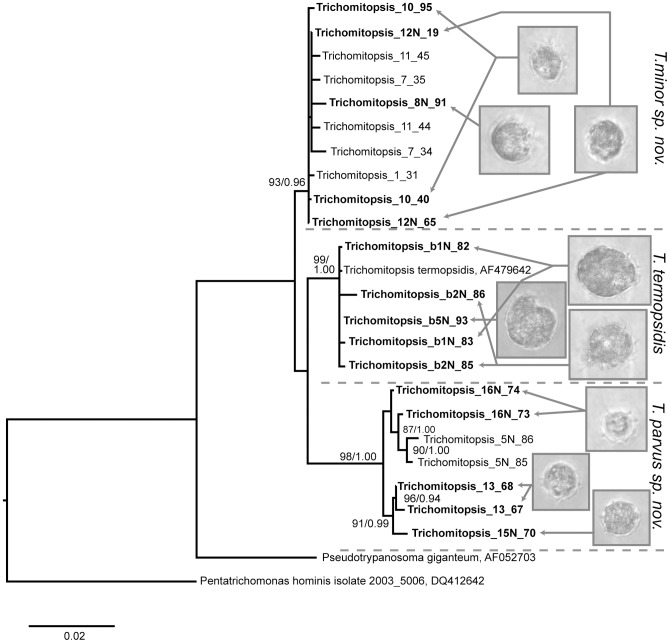
Phylogenetic tree of single-cell SSU rRNA barcodes from *Trichomitopsis* isolated from the hindgut of *Zootermopsis angusticollis*. Sequences from *Pseudotrypanosoma giganteum* (AF052703 from *Porotermes adamsoni*) and *Pentatrichomonas hominis* (DQ412642 from the preputial cavity of the domestic cattle, *Bos taurus*) were included to root the tree. The best ML tree is shown. Numbers at nodes indicate ML bootstrap support and Bayesian posterior probability values. Statistical support is shown only for nodes with >70% bootstrap support and >0.90 posterior probability.

**Figure 6:**
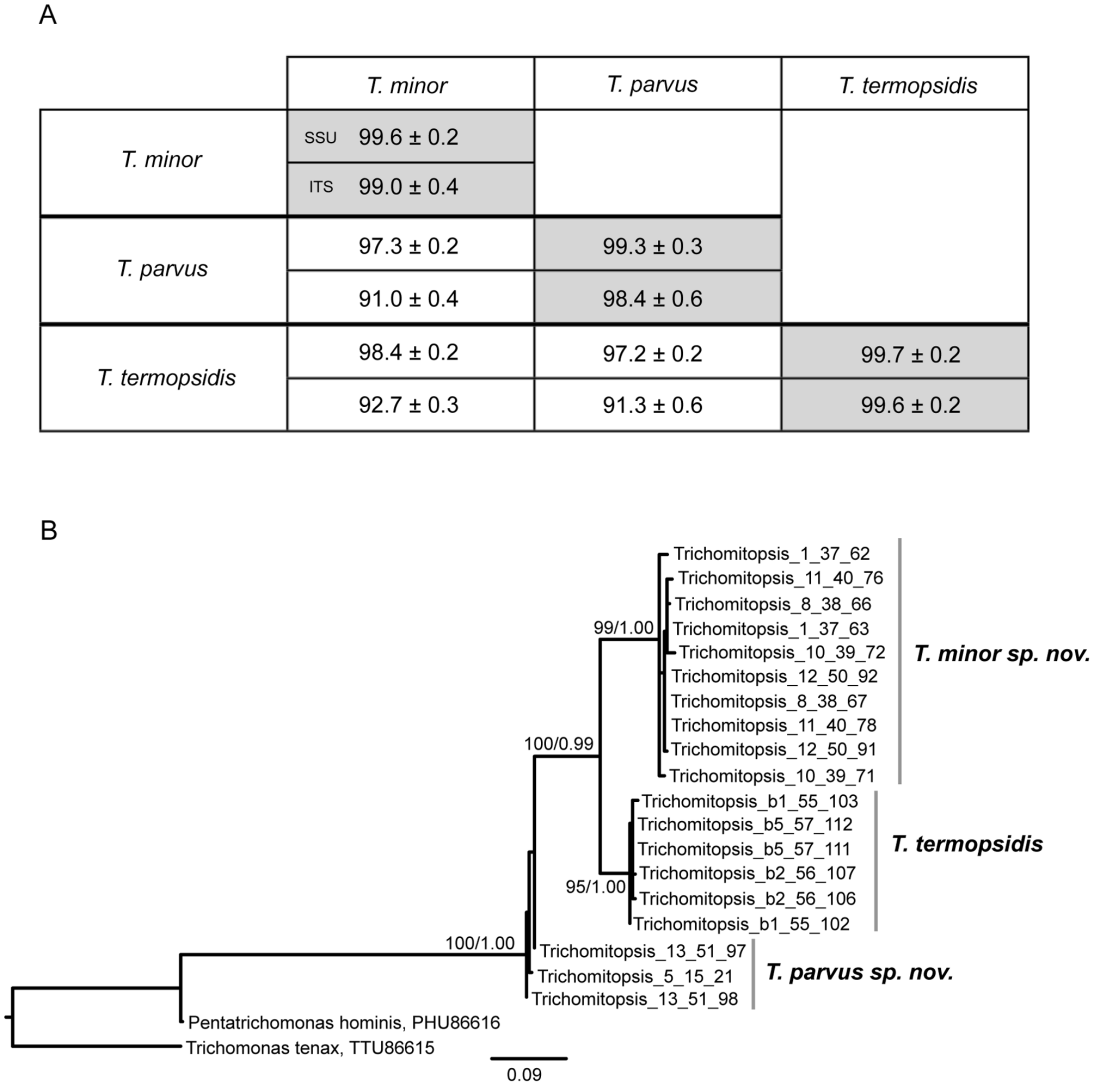
Comparing SSU and ITS barcodes from manually isolated *Trichomitopsis* cells from *Zootermopsis angusticollis.* A) Pairwise similarity matrix for DNA sequences from *Trichomitopsis* species. The upper and lower values are for SSU and ITS sequences, respectively. The mean pairwise similarities (± standard deviation) for comparisons of sequences within and between species are reported. B) Phylogenetic tree of single-cell ITS barcodes from *Trichomitopsis* isolated from the hindgut of *Zootermopsis angusticollis*. Sequences from *Pentatrichomonas hominis* and *Trichomonas tenax* were included to root the tree. The best ML tree is shown. Numbers at nodes indicate ML bootstrap support and Bayesian posterior probability values. Statistical support is shown only for nodes with >70% bootstrap support and >0.90 posterior probability.

Phylogenetic analyses of the *Trichomitopsis* SSU rRNA gene sequences resulted not in a single lineage, as expected, but rather three distinct lineages (Figure 5). All sequences from the larger morphotype shared at least 99.5% identity with the existing *T. termopsidis* sequence, which was characterized from isolated cells at the larger end of the spectrum reported [6]. Sequences from the smaller morphotype resulted in two distinct lineages, each with greater than 99% within-cluster mean pairwise similarity, and sharing less than 98.5% identity with other clusters (Figure 6A). Once again, this result was confirmed by analyses of ITS sequences, where the same three clusters were found and the same cells shown to correlate with each cluster (Figure 6B). In this case, the within-cluster sequences share on average greater than 98% similarity, whereas the mean between-cluster similarities were less than 93% (Figure 6A). Based on their morphological differences from *T. termopsidis* and the two distinct clusters that consistently form in analyses of molecular diversity from single cells, we have named these two species *Trichomitopsis parvus* sp. nov. and *Trichomitopsis minor* sp. nov. (see Taxonomic Synopsis below).

### *Trichonympha* and *Trichomitopsis* speciation

The new species of *Trichonympha*, *T. postcylindrica*, and the 3 previously described species form a monophyletic cluster exclusive of other *Trichonympha* symbionts from non-*Zootermopsis* termites. Therefore, the *Trichonympha* species in *Zootermopsis* likely diversified from a common ancestor within the hindgut after the divergence of the *Zootermopsis* lineage. *Archotermopsis*, the closest relative of *Zootermopsis*, does not host *Trichonympha* species in their hindguts, but *A. wroughtoni* does harbour a related symbiont - *Protrichonympha pristina* [2], [36]. Based on the available SSU data, the closest relatives of the *Trichonympha* species from *Zootermopsis* are *Trichonympha* from *Hodotermopsis* and *Reticulitermes* (Figure 1).

Similarly, the multiple *Trichomitopsis* species form a monophyletic group and also likely originated within the *Zootermopsis* lineage. Currently, a single species, *T. termopsidis*, is thought to commonly occur in the hindguts of all 3 species of *Zootermopsis*. However, a closer examination of the hindguts of *Z. nevadensis* and *Z. laticeps* may also reveal multiple species comprising the *Trichomitopsis* morphotype. *T. termitis* from the hindgut of *A. wroughtoni* [36], [37], *T. barbouri* in *Glyptotermes angustus* [38], and *T. cartagoensis* in *G. contracticornis* [38] are the closest known relatives of the *Trichomitopsis* from *Zootermopsis,* but molecular data are not available for these species so their phylogenetic relationships are not known. The closest relative to *Trichomitopsis* for which there are molecular data is *Pseudotrypanosoma giganteum* from the hindgut of *Porotermes adamsoni*, a relative of *Zootermopsis,* and this species groups outside of the *Trichomitopsis* cluster (Figure 5).

In addition to examining species-level diversity, ITS data should provide a more variable marker to assess population level differences between *Trichonympha* and *Trichomitopsis* symbionts in *Z. angusticollis* and *Z. nevadensis*. In SSU trees, the *Z. nevadensis* symbiont sequences cluster with the corresponding *Z. angusticollis* symbiont species suggesting they are the same or closely related co-speciated symbionts. With more extensive sampling of *Zootermopsis* populations, ITS data may reveal genetic distinctions between the symbiont populations of *Z. angusticollis* and *Z. nevadensis,* and even between the subspecies *Z. nevadensis* subsp. *nuttingi and Z. nevadensis subsp. nevadensis* which have distinct geographic distributions. Given the close similarity between *Z. angusticollis* and *Z. nevadensis*, the correct identification of the host is important for symbiont identification and it will be desirable to verify the identity of the host using DNA barcodes. Ultimately, the symbionts of *Z. angusticollis* and *Z. nevadensis* provide an opportunity to examine the rate of symbiont diversification relative to that of their hosts and possible mechanisms of speciation.

Undoubtedly, better molecular sampling of *Trichonympha* and *Trichomitopsis* species and their relatives would clarify the evolutionary relationships of these symbionts with each other and with their hosts. Nevertheless, the monophyly of the *Trichonympha* and *Trichomitopsis* species from *Zootermopsis* hosts indicates that these symbionts have almost certainly diversified into multiple species within the environment of the *Zootermopsis* hindgut. This diversification is likely an example of sympatric speciation, but it is not known what factors have lead to the diversification of *Trichonympha* or *Trichomitopsis.* These species appear to be similarly distributed throughout the hindgut and comparative physiological studies do not exist. The hindgut environment, however, is not uniform and adaptation to microniches due to differences in oxygen or hydrogen concentrations is a possibility [39]. For *Trichonympha*, another possibility is their association with ecto- and endobacterial symbionts that may facilitate the separation of ecological niches within the hindgut by performing distinctive biochemical functions such as providing amino acids or other nitrogenous compounds [17], [40], [41].

### Conclusions

Accurate descriptions of diversity are essential for understanding the ecology and evolution of any biological community. Especially for microbial communities, these descriptions are lacking because most microbes cannot be easily cultivated and descriptions based solely on morphology greatly underestimate the genetic diversity. For nearly 100 years, the hindgut of *Z. angusticollis* was known to harbour 3 species of *Trichonympha* and 1 species of *Trichomitopsis*, but even for these largest symbionts, we show that species diversity has been underestimated. We used single cell isolations and DNA sequencing of molecular markers alongside morphological observations and discovered a new *Trichonympha* species, *T. postcylindrica sp. nov.* and two species of *Trichomitopsis*, *T. minor sp. nov.* and *T. parvus sp. nov.* in the hindgut of *Z. angusticollis*. This expansion of the known diversity in the hindgut of *Z. angusticollis* provides necessary knowledge to better understand the ecological interactions, diversification, evolution, and host co-evolution of these symbiotic microorganisms.

## Taxonomic Synopsis

### *Trichonympha postcylindrica* Tai and Keeling, *sp. nov*

urn:lsid:zoobank.org:act:891EAE1C-F1FC-432F-9F9D-D2EA2F93409B.

#### Type host

Zootermopsis angusticollis.

#### Type locality

N 49.2531 W 123.2113, Pacific Spirit Park, Vancouver, BC, Canada.

#### Diagnosis

Large, multi-flagellate symbiont from the hindgut of *Zootermopsis angusticolli*s. Width ranges from 55 to 78 µm, averaging 67 µm. Length ranges from 135 to 209 µm, averaging 180 µm. The average length to width ratio is 2.70. Anterior end is tapered ending in a rounded cap or rostrum. Thousands of flagella emerge over much of the cell. Single nucleus located posteriorly. Distinct from other species of *Trichonympha* based on a cylindrical extension of ectoplasm on the posterior end and by distinct SSU rRNA and ITS sequence.

#### Hapantotype

Mounted slide deposited at the Beaty Biodiversity Museum, University of British Columbia, Vancouver, Canada under the accession number MI-PR202.

#### Gene sequence

SSU rRNA GenBank accession number KC136668 (clone 3_8). ITS rRNA GenBank accession number KC136740 (clone 3_29_37).

#### Etymology

A cylindrical extension on the posterior end of the cell.

### *Trichomitopsis minor* Tai and Keeling, *sp. nov*

urn:lsid:zoobank.org:act:58E9FE9B-E42D-426C-ACA9-2928FCB8D3B7.

#### Type host

*Zootermopsis angusticollis.*

#### Type locality

N 49.2765 W 123.2270, Pacific Spirit Park, Vancouver, BC, Canada.

#### Diagnosis

Symbiont from the hindgut of *Zootermopsis angusticollis* ranging from 12 to 37 µm in diameter (averaging 25 µm) with a recurrent flagellum forming an undulating membrane and a protruding axostyle. Distinct from *Trichomitopsis termopsidis* based on its smaller size. Distinct from other *Trichomitopsis* based on SSU rRNA gene and ITS region sequences.

#### Hapantotype

Mounted slide deposited at the Beaty Biodiversity Museum, University of British Columbia, Vancouver, Canada under the accession number MI-PR202.

#### Gene sequence

SSU rRNA GenBank accession number KC136706 (clone 10_40). ITS rRNA GenBank accession number KC136762 (clone 10_39_71).

#### Etymology

Small in size.

### *Trichomitopsis parvus* Tai and Keeling, *sp. nov*

urn:lsid:zoobank.org:act:CA53646C-C81C-48C3-936A-80E973180FA5.

#### Type host

Zootermopsis angusticollis.

#### Type locality

N 49.2765 W 123.2270, Pacific Spirit Park, Vancouver, BC, Canada.

#### Diagnosis

Symbiont from the hindgut of *Zootermopsis angusticollis* ranging from 13 to 30 µm (averaging 25 µm) in diameter with a recurrent flagellum forming an undulating membrane and a protruding axostyle. Distinct from *Trichomitopsis termopsidis* based on its smaller size. Distinct from other *Trichomitopsis* based on SSUrRNA and ITS sequences.

#### Hapantotype

Mounted slide deposited at the Beaty Biodiversity Museum, University of British Columbia, Vancouver, Canada under the accession number MI-PR202.

#### Gene sequence

SSU rRNA GenBank accession number KC136710 (clone 13_68). ITS rRNA GenBank accession number KC136764 (clone 13_51_97).

#### Etymology

Small in size.

## Materials and Methods

### Single cell isolation

Late instar nymphs from *Z. angusticollis* colonies were collected from decaying logs in Pacific Spirit Park, adjacent to the University of British Columbia campus, Vancouver, Canada. The samples were collected under the Metro Vancouver Regional Parks Research Permit No. VTPAC2011. The identity of the termites was confirmed by obtaining DNA sequences from their mitochondrial COI gene as described in Booth et al. 2012 [33]. The hindgut was removed and the contents were resuspended in Trager's Medium U [42]. Using an inverted microscope and micromanipulation, single cells that were morphologically consistent with *Trichonympha* and *Trichomitopsis* species were transferred to fresh buffer 3 times, placed in a microcentrifuge tube, and stored at −20°C. Photographs were taken of all individually isolated cells.

### DNA extraction, PCR amplification, and sequencing

DNA from single cells was extracted using the MasterPure Complete DNA and RNA Purification kit (Epicentre) following the manufacturer's instructions except the extracted DNA was resuspended in 4.5 µL TE buffer. For all PCR reactions, a 25 µL reaction mix consisted of 5 pmoles each of the forward and reverse primers, 2 µL of DNA template, and 1X EconoTaq PLUS GREEN (Lucigen).

Nearly the entire SSU (18S) rRNA gene was amplified by PCR using the PF1 and FAD4 primers [21]. If this PCR did not amplify the SSU rRNA gene fragment sufficiently, a nested PCR was performed using 1 µL of the primary PCR as template and the primers GGF and GGR [21]. The primary and nested PCRs were incubated using the following thermal profile: 94°C for 2 min, 35 cycles of 94°C for 30 s, 50°C for 1 min, and 72°C for 2 min, and a final extension at 72°C for 10 min.

The ITS region was amplified using the forward primer ITSFpara (5′-GTC CCT GCC CTT TGT ACA CAC C-3′) modified from [43], and the reverse primer NC2 (5′-TTA GTT TCT TTT CCT CCG CT-3′) [44]. These primers anneal to the 3′ end of the SSU rRNA gene and the 5′ end of the large subunit (LSU, 28S) rRNA gene, respectively. The PCR conditions were: 94°C for 2 min, 35 cycles of 94°C for 30 s, 54°C for 30 s, and 72°C for 1 min, and a final extension at 72°C for 5 min.

For *Trichonympha* cells, a larger portion of the SSU rRNA gene was amplified with the ITS region using TrichoSSUmidF (5′-CGA GAC TAC CGC CAA ATA-3′) and NC2. TrichoSSUmidF is a *Trichonympha*-specific primer that anneals in the middle of the SSU rRNA gene approximately 400 bp away from the 3′ end. This larger DNA fragment was amplified in order to have sufficient SSU rRNA sequence data to distinguish the *Trichonympha* species. The PCRs were incubated at 94°C for 2 min, followed by 35 cycles of 94°C for 30 s, 52°C for 1 min, and 72°C for 1 min, and a final extension at 72°C for 10 min.

All PCR products were ligated into plasmid vectors and cloned using the Strataclone PCR cloning kit (Agilent Technologies) following the manufacturer's protocol. Plasmid DNA was extracted from positive clones using the FastPlasmid Mini Kit (5 Prime). For each single *Trichonympha* or *Trichomitopsis* cell, two clones of the SSU rRNA gene fragment and two clones of the ITS region were Sanger sequenced (NAPS facility, UBC) from both strands with the BigDye Terminator kit v. 3.1 (Applied BioSystems). In a few rare cases, only a single clone was sequenced. All of the sequences have been deposited in GenBank under accession numbers KC136612-KC136766.

### Phylogeny

Separate alignments were used to calculate the phylogeny for the SSU rRNA sequences from *Trichonympha* and *Trichomitopsis*. Related parabasalid SSU rRNA sequences were obtained from GenBank and aligned to the *Trichonympha* or *Trichomitopsis* sequences using MAFFT [45] from an online server (http://www.ebi.ac.uk/Tools/msa/mafft/) with the default settings ( =  L-INS-i). The ends of the alignments were trimmed manually. Gblocks was used to remove highly variable and ambiguously aligned sites, but allowing smaller final blocks, gap positions, and less strict flanking positions (http://molevol.cmima.csic.es/castresana/Gblocks_server.html) [46]. Phylogenetic trees were calculated from maximum likelihood (ML) analysis using RAxML 7.0.4 [47] and Bayesian analysis using MrBayes 3.2 [48]. The ML analyses implemented a general time reversible (GTR) model of nucleotide substitution with the gamma model of rate heterogeneity. Statistical support for the consensus tree was assessed from 1000 bootstrap replicates. The Bayesian analyses also used a GTR + gamma model. For the *Trichonympha* alignment, 4 chains were sampled every 100 generations from 2 runs for 1 500 000 generations. Diagnostics were run every 1000 generations with a relative burnin of 25% of the tree samples. After 1 500 000 generations, the average standard deviation of the split frequencies from the 2 runs was less than 0.01. The *Trichomitopsis* Bayesian analysis was run for 1 000 000 generations.

The ITS sequences from *Trichonympha*, *Trichomitopsis*, and representative parabasalid ITS sequences from GenBank were used to calculate phylogenetic trees using ML and Bayesian analyses as described above. The Bayesian analyses were run for 1 000 000 generations for both *Trichonympha* and *Trichomitopsis* ITS alignments.

### Topology test

The approximately unbiased (AU) test was used to assess the confidence of all possible branching topologies in the phylogeny of the 4 distinct clusters of*Trichonympha* from *Z. angusticollis*. Given 4 clusters of *Trichonympha*, there are 15 possible topologies for these clusters. For both SSU and ITS data, each of these topologies was generated by editing the branching order from the best ML tree in TreeView [49]. RAxML 7.0.4 [47] was used to generate per-site log likelihoods from the SSU and ITS alignments for each topology and CONSEL [50] was used to conduct the AU test.

### Nomenclatural Acts

The electronic edition of this article conforms to the requirements of the amended International Code of Zoological Nomenclature, and hence the new names contained herein are available under that Code from the electronic edition of this article. This published work and the nomenclatural acts it contains have been registered in ZooBank, the online registration system for the ICZN. The ZooBank LSIDs (Life Science Identifiers) can be resolved and the associated information viewed through any standard web browser by appending the LSID to the prefix “http://zoobank.org/”. The LSID for this publication is: urn:lsid:zoobank.org:pub:EF0EDBC0-9C8F-4B2F-8B62-9C748691B5D3. The electronic edition of this work was published in a journal with an ISSN, and has been archived and is available from the following digital repositories: PubMed Central, LOCKSS.

## Supporting Information

Table S1 **P-values from AU tests on all possible tree topologies of the 4** ***Trichonympha*** **clusters.**
(DOC)
