# Supplementary material for: Single-Cell DNA Barcoding Using Sequences from the Small Subunit rRNA and Internal Transcribed Spacer Region Identifies New Species of Trichonympha and Trichomitopsis from the Hindgut of the Termite Zootermopsis angusticollis
Source: PLoS One. 2013 Mar 11;8(3):e58728. doi: 10.1371/journal.pone.0058728 (PMC3594152; doi:10.1371/journal.pone.0058728)
Supplement: Table S1 — P-values from AU tests on all possible tree topologies of the 4 Trichonympha clusters. (DOC) [file pone.0058728.s001.doc]

**Table S1.** **P-values from AU tests on all possible tree topologies of the 4 *Trichonympha* clusters.**  * indicates topologies with p-values less than 0.05 that can be rejected. The topology of the best ML tree from the SSU phylogeny is topology 1 and from the ITS phylogeny is topology 2. Neither of these was rejected by the AU tests.

| Topology | SSU p-value | ITS p-value |
| --- | --- | --- |
| 1 (topology from best SSU ML tree) | 0.888 | 0.165 |
| 2 (topology from best ITS ML tree) | 0.173 | 0.775 |
| 3 | 0.116 | 0.118 |
| 4 | 0.033* | 0.120 |
| 5 | 0.176 | 0.595 |
| 6 | 0.301 | 0.595 |
| 7 | 0.316 | 0.118 |
| 8 | 0.100 | 0.094 |
| 9 | 0.271 | 0.094 |
| 10 | 0.025* | 0.094 |
| 11 | 0.064 | 0193 |
| 12 | 0.038* | 0.126 |
| 13 | 0.098 | 0.192 |
| 14 | 0.065* | 0.178 |
| 15 | 0.443 | 0.166 |
